# Canine carcinomas in benign mixed tumours: versican expression and association with invasion

**DOI:** 10.1186/1753-6561-7-S2-P39

**Published:** 2013-04-04

**Authors:** Karine A Damasceno, Angélica C Bertagnolli, Alessandra Estrela-Lima, Lorena GR Ribeiro, Bruna S Rabelo, Cecília B Campos, André LB Barros, Geovanni D Cassali

**Affiliations:** 1 Department of General Pathology, Biological Sciences Institute, Universidade Federal de Minas Gerais, Belo Horizonte, MG, Brazil; 2 Fepagro Animal Health - Desidério Finamor Institute of Veterinary Research (IPVDF), Eldorado do Sul, RS, Brazil; 3 Department of Pathology and Clinics, School of Veterinary Medicine and Zootechny, Universidade Federal da Bahia, Salvador, BA, Brazil; 4 Department of Clinical and Toxicological Analyses, School of Pharmacy, Universidade Federal de Minas Gerais, Belo Horizonte, MG, Brazil

## Background

Components of the extracellular matrix have been studied in the attempt to elucidate the mechanisms involved with biological behavior of tumours. The presence of the proteoglycan versican has been strongly associated to cancer development and progression, however its relationship with invasion and tumoral progression has been little studied in veterinary medicine. Carcinomas in benign mixed tumours (CBMT) are one of the most common malignant tumour in female dogs and can serve as a model for studies on tumour progression. The aim of this paper was to evaluate the expression of versican in *in situ* and invasive carcinomatous areas of CBMT.

## Materials and methods

Immunohistochemical staining for versican and confirmation of invasion areas with staining for p63 and smooth muscle α-actin (α-SMA) were performed on 49 cases of CBMT.

## Results

Invasion was considered when suspicious Haematoxylin-Eosin stained areas revealed a total loss of immunoreactivity for α-SMA and p63. Versican immunoreactivity was less intense adjacent to *in situ* carcinomatous regions when compared to invasive regions in which staining was found as more extensive areas with strong expression.

## Conclusions

Obtained data reveal that, in carcinomas in benign mixed tumors, versican expression differs significantly within invasive and *in situ* areas, suggesting the role of this molecule in tumor progression.

## Financial support

FAPEMIG, CNPq and CAPES.

